# Healthcare Professionals Promotion of Physical Activity with Older Adults: A Survey of Knowledge and Routine Practice

**DOI:** 10.3390/ijerph18116064

**Published:** 2021-06-04

**Authors:** Conor Cunningham, Roger O’Sullivan

**Affiliations:** 1 Institute of Public Health, Belfast BT1 4JH, UK; Roger.OSullivan@publichealth.ie; 2 Bamford Centre for Mental Health and Wellbeing, Ulster University, Belfast BT37 0QB, UK

**Keywords:** physical activity, healthcare professionals, older adults, theoretical domains framework, policy, behaviour change

## Abstract

Healthcare professionals have a key role in promoting physical activity, particularly among populations at greatest risk of poor health due to physical inactivity. This research aimed to develop our understanding of healthcare professionals knowledge, decision making and routine practice of physical activity promotion with older adults. A cross-sectional survey was conducted with practicing healthcare professionals in general practice, physiotherapy, occupational therapy and nursing in Ireland and Northern Ireland. We received 347 eligible responses, with 70.3% of all respondents agreeing that discussing physical activity is their job and 30.0% agreeing that they have received suitable training to initiate conversations with patients about physical activity. Awareness of the content and objectives of national guidelines for physical activity varied considerably across the health professions surveyed. Less than a third of respondents had a clear plan on how to initiate discussions about physical activity in routine practice with older adults. Assessment of physical activity was not routine, neither was signposting to physical activity supports. Considering the COVID-19 pandemic and its implications, 81.6% of all respondents agreed that healthcare professionals can play an increased role in promoting physical activity to older adults as part of routine practice. Appropriate education, training and access to resources are essential for supporting healthcare professionals promotion of physical activity in routine practice. Effective physical activity promotion in healthcare settings has the potential for health benefits at a population level, particularly in older adult populations.

## 1. Introduction

The health benefits of physical activity for older adults are well established [1]. There is strong evidence that physical activity contributes to increased physical function, reduced impairment, independent living, and improved quality of life in both healthy and frail older adults [2]. International guidelines recommend that all older adults (65+ years) should aim to do at least 150–300 min of moderate intensity or 75–150 min of vigorous intensity aerobic activity throughout the week, with muscle strengthening and multicomponent balance training on 2 or more days per week [3]. In addition to recommending that all older adults should undertake regular physical activity, these guidelines also emphasize the benefits to older adults of ‘moving more’ and sitting less throughout the day, as doing some physical activity is better than none [3]. However, for many older adults, ageing is defined by rapid declines in levels of physical activity, loss of mobility and functional independence, and premature morbidity [4]. Therefore, this stage of life represents an important period for promoting physical activity to improve functions of daily living and slow progression of disease and disability [5].

Effective national action to reverse trends in inactivity across the life course requires a ‘systems-based’ approach, with action across different sectors [6]. As part of this approach, healthcare services can play an important role by implementing systems for patient assessment and counselling, and by strengthening the provision of and access to opportunities and programmes that enable older adults to increase their levels of physical activity [7]. Healthcare professionals play a central role in the promotion of a variety of health behaviours and are ideally positioned to promote physical activity [8]. Evidence suggests that they can positively impact patient behaviour by routinely assessing physical activity levels and by using brief practical interventions (advice or counselling on how to initiate and maintain healthy behaviours) with links to community-based support for behaviour change [9]. These are key actions in promoting health enhancing physical activity to reduce the incidence of chronic disease and/or to manage a range of chronic conditions and weight maintenance [10]. Indeed, recent healthcare policy developments on the island of Ireland (Ireland and Northern Ireland) recognize that preventing and reducing chronic disease across the life course by addressing inactivity (amongst other modifiable risk factors) requires a cultural shift on improving health and wellbeing through strategies that focus on health promotion and disease prevention [11].

Enhancing our understanding of current practice in relation to physical activity promotion in the health services is crucial to inform the development of evidence-based strategies for improving uptake of policy into practice. Previous research suggests that healthcare professionals instigate brief physical activity interventions opportunistically in a quarter of appropriate instances [9] and there is a disparity between the development of guidelines for physical activity and their dissemination and integration into routine clinical practice for many healthcare professionals [12,13,14]. Barriers to physical activity promotion have been reported by healthcare professionals, including lack of awareness, expertise, and lack of time and incentive [8]. It is unclear what level of knowledge around physical activity exists across a broad range of healthcare professionals on the island of Ireland, and to what extent physical activity promotion with older adults is involved in decision making in routine practice. This research is therefore focused on developing our understanding of healthcare professionals approaches to promoting physical activity to older adults in Ireland and Northern Ireland by evaluating their knowledge of current physical activity guidelines and exploring the factors which may influence their clinical judgements and decision making in promoting physical activity to older adults in routine practice. As this research took place during the COVID-19 pandemic, healthcare professionals perceptions of the implications of the public health and social measures for older adults’ levels of physical activity, and their resultant behaviour(s) related to physical activity promotion with older adults in routine practice were also explored.

## 2. Materials and Methods

### 2.1. Sample and Eligibility

This was a cross-sectional study of practicing healthcare professionals in general practice, physiotherapy, occupational therapy and nursing in Ireland and Northern Ireland in 2020. Healthcare professionals who were not registered to practice in one of these four professional groups, those who were retired, working outside of Ireland or Northern Ireland, or who did not have clinical contact with older adults (defined as ≥65 years) as part of routine care were excluded from the study.

### 2.2. Survey Tool

A 43-item (3-section) survey was developed using the theoretical domains framework (TDF). The TDF is an integrative framework of theories of behaviour change developed to identify influences on healthcare professional behaviour in the implementation of evidence-based recommendations [15]. It has been used extensively to identify barriers and facilitators for individual uptake of evidence-based practices and for implementation design and research [16].

Section 1 of the survey captured demographic and employment data as well as healthcare professionals self-reported levels of physical activity, knowledge of physical activity guidelines and awareness of resources to facilitate knowledge and practice development. Section 2 assessed TDF domains of healthcare professional’s behaviour in assessment, discussion, and prescription of physical activity in routine practice. Healthcare professionals perceptions of the implications of the COVID-19 pandemic on older adults’ levels of physical activity and views on the role of physical activity promotion to older adults in light of the pandemic were also examined. Section 3 of the survey used clinical vignettes (3 for each healthcare profession) to enable healthcare professionals to self-report routine practice in relation to physical activity promotion with older adults. Analysis and findings for Section 3 of the survey are reported in a separate publication.

### 2.3. Survey Piloting and Procedure

A research project advisory group (*N* = 10) participated in pilot testing, refinement, and approval of the survey. This group included appointed representatives of the Royal College of General Practitioners in Northern Ireland and the Irish College of General Practitioners in Ireland; the Chartered Society of Physiotherapy in Northern Ireland and the Irish Society of Chartered Physiotherapists in Ireland; the Royal College of Nursing in Northern Ireland and the Department of Public Health Nursing in Ireland; and the Royal College of Occupational Therapists in Northern Ireland and the Association of Occupational Therapists Ireland. The survey was live for a 2-month period from mid-August to mid-October 2020, during which time the link to the survey was promoted through focused email distribution and promoted widely on social media.

### 2.4. Data Analysis

All eligible returned surveys were included in the analysis regardless of missing data; consequently, the number of total responses for each survey item is varied. Descriptive and explorative analysis of the data were performed using IBM SPSS V.24 (IBM Inc., Armonk, NY, USA). Descriptive statistics were used to describe data from the survey reporting frequencies of responses. Pearson’s chi squared tests were used to compare knowledge of guidelines for physical activity with assessment, discussion, prescription, and signposting of physical activity in routine practice. Knowledge of the guidelines was classified by dividing the participants into two groups: (1) those who correctly recalled three specific components of the physical activity guidelines for older adults (number of minutes per week of moderate intensity physical activity, vigorous intensity physical activity, and the number of days of strength, balance, and flexibility activities recommended for optimal health benefits); and (2) those who indicated that they did not know the guidelines, or who incorrectly recalled one or more specific component of physical activity guidelines for older adults. Statistical significance was set at *p* value <0.05.

## 3. Results

### 3.1. Participant Characteristics

In total, 573 responses were received. Of these, 143 did not meet the inclusion criteria, and a further 83 did not complete components of the survey required to be included in subsequent analysis. A total of 347 respondents met the inclusion criteria and their responses were included in subsequent analysis. Participant characteristics are presented in Table 1. Of the respondents, 44 (12.7%) were male and 299 (86.2%) were female. Three quarters of respondents (74.4%) were healthcare professionals practicing in Ireland. Nearly half of all respondents (49.0%) were physiotherapists. The proportion of all respondents who reported that they had 26+ years of practice experience was 27.4% (*n* = 95). Most respondents worked in the public sector (80.7%, *n* = 280). The proportion of respondents who achieved the recommended level of moderate intensity physical activity over a week was 40.6% (*n* = 141).

### 3.2. Knowledge, Understanding and Use of Physical Activity Guidelines

Responders were asked how aware they were of the content and objectives of national guidelines in their jurisdiction. Of all respondents, 42.7% (*n* = 148) (62.9% of physiotherapists, 34.2% of nurses, 22.2% of general practitioners and 19.4% of occupational therapists) agreed that they were aware of the content and objectives of national guidelines for physical activity in their jurisdiction (Table 2). Of all respondents, 35.4% (*n* = 123) agreed that they were aware of the content and objectives of national guidelines for physical activity for older adults in their jurisdiction. The percentage of those that ‘agreed’ varied considerably across the healthcare professions surveyed (Table 2).

Responders were also asked about their knowledge of three specific components of physical activity recommendations for older adults (number of minutes per week of moderate intensity physical activity, vigorous intensity physical activity, and the number of days of strength, balance, and flexibility activities recommended for optimal health benefits). Of all respondents, 61.1% (*n* = 212) reported that they knew how many weekly minutes of moderate intensity physical activity were recommended for older adults, and 38.6% (*n =* 134) of all respondents reported that they knew how many minutes of weekly vigorous intensity physical activity were recommended for older adults. However, when prompted, 49% (*n =* 170) correctly recalled the number of minutes of moderate intensity activity, and 27.4% (*n =* 95) correctly recalled the number of minutes of vigorous activity. 54.2% (*n =* 188) of all respondents reported that they knew how many days per week older adults were recommended to perform strength, balance, and flexibility training. The proportion who correctly recalled the number of days when prompted was 51.6% (*n =* 179). Of all respondents, 24.5% (*n =* 85) correctly recalled all three specific components of physical activity guidelines for older adults (Table 2).

Most respondents (64.6%) agreed that physical activity guidelines have a place in routine practice. However, only 26.5% of physiotherapists, 15.8% of nurses, 14.0% of occupational therapists, and 5.6% of general practitioners agreed that there is sufficient time allocated to implement physical activity guidelines for adults/older adults in day-to-day work (Table S1).

### 3.3. Awareness of Resources

**Figure 1:**
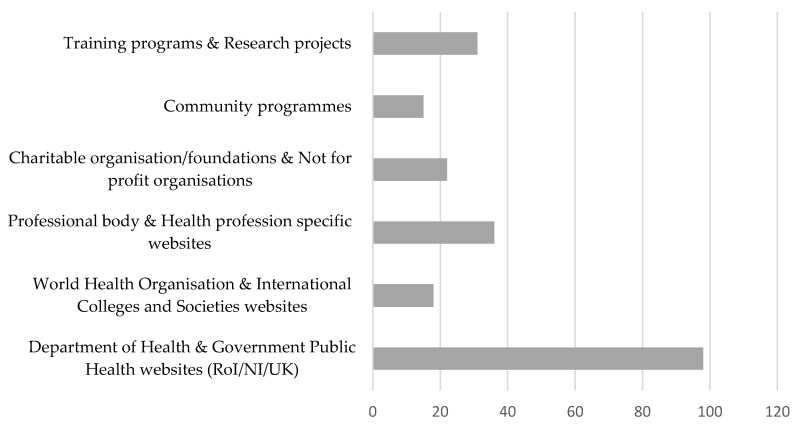
Resources accessed by participants for knowledge development of physical activity promotion.

Of all respondents, 47.6% (*n =* 165) (64.1% of physiotherapists, 47.4% of nurses, 33.0% of occupational therapists and 11.1% of general practitioners) reported that they were aware of resources to facilitate their knowledge development and practice of assessment/discussion/prescription of physical activity with patients as a part of routine care. The most frequently cited resources included government health department websites, professional body websites and health profession specific websites, and training programmes and research projects with online physical activity resources and toolkits (Figure 1).

### 3.4. Theoretical Domains of Health Professionals Behaviour

Table S1 shows the theoretical domains of healthcare professional’s behaviour in assessment, discussion, and prescription of physical activity in routine practice.

#### 3.4.1. Assessment of Patient’s Physical Activity Levels

Responders were asked about their use of screening tools to measure patients’ physical activity levels. Nearly half of all respondents (48.7%, *n =* 169) reported that they ‘never’ formally assess whether a patient is active or inactive as part of routine practice. The proportion who reported that they ‘sometimes’, ‘usually’, or ‘always’ assess whether a patient is active or inactive was 40.0% (*n =* 139). A variety a functional assessment tools (e.g., the Timed Up and Go (TUG) test, 6-min walk test), questionnaire based (e.g., General Practice Physical Activity Questionnaire (GPPAQ)) and device-based measures of physical activity (e.g., pedometers) were reported. Having knowledge of three specific components of physical activity guidelines for older adults was significantly associated with formally assessing whether a patient is active or inactive as part of routine practice (Table S1).

#### 3.4.2. Discussing Physical Activity with Patients

Responders were asked if they considered it a part of their professional role to promote physical activity to patients. Most respondents (70.3%, *n =* 244) agreed that discussing physical activity with patients was part of their work as a healthcare professional. Many (74.1%, *n =* 257) ‘agreed’, or ‘somewhat agreed’ that it was easy to remember to discuss physical activity with patients (74.1%, *n =* 257) and that they were confident that they could discuss physical activity as part of routine practice even when the patient was not motivated (72%, *n =* 250) or when there was little time (63.8%, *n =* 221). Overall, 30.0% of all respondents (*n =* 104) agreed that they had received suitable training to initiate conversations with patients about physical activity. This percentage was higher for physiotherapists (44.1%) (Table S1). We found that 36.1% of general practitioners, 21.4% of occupational therapists, and 23.7% of nurses did not agree that they have received suitable training to initiate conversations with patients about physical activity.

#### 3.4.3. Discussing Physical Activity with Older Adults

Most respondents (68.0%, *n =* 236) agreed that discussing physical activity with older adults was part of their work as a health professional, with the majority ‘agreeing’, or ‘somewhat agreeing’ that it is something they do automatically (67.6%, *n =* 233) and is useful (82.4%, *n =* 286). Many ‘agreed’, or ‘somewhat agreed’ that they were aware of how to initiate conversations about physical activity with older adults (83.5%, *n =* 290), that they had the skills to initiate conversations with older adults about physical activity (80.1%, *n =* 278) and that it was something that they typically did within their organization (72.1%, *n =* 250). There was a significant association between having knowledge of three specific components of physical activity guidelines for older adults and initiating conversations with patients about physical activity as part of routine practice (Table S1). However, even though many respondents agreed that that they intended to discuss physical activity in their next consultation/appointment with an older adult as part of routine care (55.0%, *n =* 191), fewer agreed that they had a clear plan on how to initiate discussions about physical activity in routine practice with older adults (30.5%, *n =* 106).

#### 3.4.4. Physical Activity Prescription and Signposting

Responders were asked how often they refer/signpost patients through referral programmes or community-based schemes. We found that 12.1% of respondents ‘always’ signposted patients to other physical activity services (i.e., exercise referral programmes/community-based physical activity initiatives). Having knowledge of three specific components of physical activity guidelines for older adults was significantly associated with signposting patients to other physical activity services as part of routine practice (Table S1). Most physiotherapists (64.1%), occupational therapists (63.1%), general practitioners (61.2%), and 50.0% of nurses reported that they ‘sometimes’ or ‘usually’ signposted patients to other physical activity services. The physical activity services that they ‘sometimes’, ‘usually’, or ‘always’ signposted to patients included exercise referral programmes; community-based active retirement groups; community-based healthcare professional follow-up (e.g., fall prevention groups, community physiotherapy/occupational therapy services); online self-management groups/virtual classes and resources. Most respondents (68.6%) agreed that assessing/discussing/prescribing physical activity with an older adult as part of routine practice would benefit the public health agenda. 23.3% of occupational therapists, 21.1% of nurses and 2.8% of general practitioners agreed that they were supported to use physical activity assessment/discussions/prescription in everyday practice. This number was higher for physiotherapists (38.8%).

### 3.5. Older Adults’ Physical Activity and the COVID-19 Pandemic

To explore healthcare professionals understanding of the potential impact of public health restrictions on older adults’ levels of physical activity, and their resultant behaviour(s) related to physical activity promotion with older adults in routine practice, the following questions were asked (see Table 3). The proportion of all respondents who indicated that the public health and social measures introduced to prevent the spread of COVID-19 had reduced older adults’ levels of physical activity was 71.2%, and considering this, 81.6% of all respondents agreed that healthcare professionals can play an increased role in promoting physical activity to older adults. Overall, 84.7% stated that they were ‘more likely’ (47.8%), or the ‘same as usual’ (36.9%) to discuss physical activity with older adults as part of routine practice considering the COVID-19 pandemic and its implications.

## 4. Discussion

Healthcare professionals play a pivotal role in educating patients about the benefits of being more active and motivating their patients to engage in a more active lifestyle [18,19]. In this study most respondents agreed that discussing physical activity is their job (TDF domain: Social/Professional Role and Identity), and that it is easy to remember to do (TDF domain: Memory, Attention & Decision processes). However, the majority had not received suitable training in initiating discussions about physical activity with patients (TDF domain: Skills). Our survey also shows that many healthcare professionals are unaware of current guidelines for physical activity in older adults (TDF domain: Knowledge)—one in four correctly answered questions about the content of these guidelines, and less than a third of respondents had a clear plan on how to initiate discussions about physical activity in routine practice with older adults (TDF domain: Behavioural Regulation).

The results of this survey have identified a range of theoretical domains that can be targeted to support healthcare professionals in their role of promoting active lifestyle change with patients. In particular, the domains of Knowledge, Skills and Behavioural Regulation were identified. These domains map directly onto the ‘Capability’ component of the COM-B model of behaviour change [20]. Building healthcare professionals’ ‘Capability’ to promote physical activity in routine practice through ‘Knowledge’ development: appropriate education on guidelines for physical activity in prevention and treatment of disease is essential. In this study, having a detailed knowledge and recall of physical activity guidelines for older adults was associated with formal assessment, initiating discussion, and referral/signposting to physical activity services as part of routine practice. Building capability through ‘Skill’ development: relevant training on initiating discussions/brief interventions to support patients with behavioural change (perhaps through motivational interviewing) is equally important. Evidence suggests that brief practical interventions by clinicians may improve short and long-term engagement with active lifestyles [21] and that components of motivational interviewing are central to this approach as a theory consistent and evidence-based technique to strengthen an individual’s motivation for change [22].

The recently introduced Making Every Contact Count (MECC) strategy places the responsibility for providing brief (and opportunistic) interventions on all healthcare professionals who may have patient contact in Ireland and Northern Ireland. By integrating MECC at the initial/undergraduate level it is planned that brief interventions will become central to many consultations [23]. In this survey most respondents were fully qualified with regular patient contact for more than 6 years, which suggests that continuing education/professional development on physical activity promotion will be crucial to maximise their impact on and support for sustained behaviour change with their patients. This finding is consistent with other studies that have highlighted the need for postgraduate training for healthcare professionals to address health behaviour change [23,24], but also the need to integrate physical activity training and its relationship with health at an undergraduate level. Several reports indicate that this issue is now receiving greater attention in undergraduate curricula [25,26].

Many healthcare professionals surveyed never formally assess whether a patient is active or inactive as a part of routine practice. In part, this may reflect the level of training and support that healthcare professionals surveyed have received on physical activity promotion broadly, and on physical assessment more specifically. The routine assessment of physical activity (and sedentary behaviour) in the health system is the basis for the surveillance of physical inactivity as a risk factor [27]. Assessing a patient’s level of physical activity can provide a valuable insight into health status and is an essential first step that can lead to important intervention opportunities, if appropriate. Those who reported that they assess physical activity and/or functional status in older adults used a variety of tools and resources. Whereas the use of tools to support the systematic screening and delivery of brief interventions is recommended, some have advocated the use of a standardised physical activity tool as a ‘vital sign’ in patients’ consultations, that should be a standard of care for all patient visits with the potential to highlight inactivity and prompt a brief intervention (counselling or referral) [28].

Few respondents ‘always’ signposted patients to other physical activity services (i.e., exercise referral programmes/community-based physical activity initiatives). The majority reported that they did this ‘usually’ or ‘sometimes’. Raising awareness of community resources (passive or active signposting) or prescribing activity as a means of referral is seen as a ‘formal’ acknowledgement of the problem (inactivity) and provides both a legitimacy to the issue and an opportunity to do something about it. Previous research has suggested that patients ‘like’ the option of being connected to resources on specific physical activity opportunities by a health professional, to consider and potentially follow up on [29].

How the topic is raised and linked to a patient’s specific health conditions is central to patient acceptance to the topic [29], highlighting again the need for specific and ongoing education and training on initiating discussions and delivering brief interventions as part of routine care. Research suggests that the ‘motivation’ provided by a healthcare professional is key to whether a patient accepts offers of being signposted to community physical activity opportunities [30]. The potential for patients to meet with people in similar circumstances from the wider community to engage in physical activity can provide an opportunity to build relationships and a supportive solution to increase activity levels [31]. Social support is important for the adoption and maintenance of physical activity, particularly for older adults [32].

The role that healthcare professionals play in promoting physical activity as part of routine care, and the support that they provide for older adults will be increasingly important in addressing the fall-out from COVID-19 on older adult’s health. It is likely that the proportion of the older adult population inactive and at risk from disease and disorders related to inactivity will have increased [33]. Those who are socio-economically disadvantaged, frail, living with multimorbidity or disability or living in residential care, may have been disproportionately affected [34].

### Strengths and Limitations

This study captured views from a diverse range of healthcare professions. To the authors’ knowledge, this is the first study to have completed this type of analysis across the island of Ireland. The TDF was utilised as an evidence-based method for identifying individual and organisational determinants of health professionals’ behaviour and decision-making. In this context, questions used within the questionnaire had established content validity and extensive piloting and pre-development work, which with the input of an expert advisory group, improved the overall validity of the final survey. The questionnaire was distributed through professional body networks across the island of Ireland and the number of valid responses (conceivably affected by the COVID-19 pandemic, during which healthcare professionals were facing unprecedented demands on their time) compares favourably to other studies conducted with healthcare professionals. However, consideration should still be given to the generalisability of the survey findings. Selection bias is an issue that needs to be considered in this context, as it is possible that healthcare professionals who are interested in and utilise physical activity in routine practice were more motivated to participate. The smaller number of respondents from general practice and nursing (relative to physiotherapy and occupational therapy) is also a potential limitation of the research.

## 5. Conclusions

Healthcare professionals have a key role in the promotion of physical activity as part of a whole-systems approach. This is highlighted in the strategic objectives of National and International Policy.

This study has shown that healthcare professionals consider it a part of their role to discuss physical activity, and many reported that it was feasible to initiate discussions about physical activity even in the face of commonly reported barriers (little time available/patient not motivated).

Successful implementation of physical activity promotion in routine practice will have substantial health benefits at a population level, particularly for older adults who stand to benefit the most from increasing levels of activity. However, continuing education and training is essential to support healthcare professionals’ knowledge and skill development if they are to be successful in this role.

## Figures and Tables

**Table 1:** Characteristics of survey participants.

	All Respondents		General Practice		Physiotherapy		Occupational Therapy		Nursing	
	*N*	%	*N*	%	*N*	%	*N*	%	*N*	%
**Professional Affiliation**										
General practitioner	36	10.4								
Physio-therapist	170	49.0								
Occupational therapist	103	29.7								
Nurse	38	11.0								
**Gender**										
Male	44	12.7	14	38.9	22	12.9	8	7.8		
Female	299	86.2	20	55.6	147	86.5	95	92.2	37	97.4
Prefer not to say	4	1.2	2	5.6	1	0.6			1	2.6
**Years qualified**										
0–5	39	11.2			18	10.6	21	20.4		
6–10	52	15.0	4	11.1	24	14.1	22	21.4	2	5.3
11–15	59	17.0	5	13.9	31	18.2	19	18.4	4	10.5
16–20	49	14.1	6	16.7	30	17.6	10	9.7	3	7.9
21–25	53	15.3	7	19.4	32	18.8	8	7.8	6	15.8
26+	95	27.4	14	38.9	35	20.6	23	22.3	23	60.5
**Healthcare setting**										
Primary	128	36.9	27	75	61	35.9	32	31.1	8	21.1
Secondary	62	17.9	7	19.4	32	18.8	17	16.5	6	15.8
Other	157	45.2	2	5.6	77	45.3	54	52.4	24	63.2
**Health sector**										
Public	280	80.7	33	91.7	141	82.9	83	80.6	23	60.5
Private	46	13.3	3	8.4	23	13.5	13	12.6	7	18.4
Other	21	6.1			6	3.5	7	6.8	8	21.1
**Region**										
Northern Ireland	89	25.6	14	38.9	59	34.7	10	9.7	6	15.8
Ireland	258	74.4	22	61.1	111	65.3	93	90.3	32	84.2
Total	347	100.0	36	100.0	170	100.0	103	100.0	38	100.0
Physical activity levels *										
Active	141	40.6	18	50.0	76	44.7	32	31.1	15	39.5
Inactive	205	59.1	17	47.2	94	55.3	71	68.9	23	60.5
Total	346	99.7	35	97.2	170	100	103	100	38	100

* Measured by Single Item Metric [17].

**Table 2:** Participant’s awareness of physical activity guidelines and resources.

Survey Question *	Answer	All Respondents		General Practice		Physiotherapy		Occupational Therapy		Nursing	
		*N*	%	*N*	%	*N*	%	*N*	%	*N*	%
I am aware of the content and objectives of national guidelines for physical activity in my jurisdiction	Agree	148	42.7	8	22.2	107	62.9	20	19.4	13	34.2
	Somewhat agree	111	32.0	12	33.3	35	20.6	44	42.7	20	52.6
	Neither agree nor disagree	13	3.7	3	8.3	3	1.8	5	4.9	2	5.3
	Somewhat disagree	19	5.5	1	2.8	4	2.4	13	12.6	1	2.6
	Disagree	20	5.8	6	16.7	2	1.2	12	11.7		
	Not stated	36	10.4	6	16.7	19	11.2	9	8.7	2	5.3
I am aware of the content and objectives of national guidelines for physical activity for older adults in my jurisdiction	Agree	123	35.4	8	22.2	89	52.4	17	16.5	9	23.7
	Somewhat agree	124	35.7	7	19.4	52	30.6	43	41.7	22	57.9
	Neither agree nor disagree	17	4.9	4	11.1	3	1.8	7	6.8	3	7.9
	Somewhat disagree	24	6.9	4	11.1	5	2.9	14	13.6	1	2.6
	Disagree	23	6.6	7	19.4	2	1.2	13	12.6	1	2.6
	Not stated	36	10.4	6	16.7	19	11.2	9	8.7	2	5.3
**Awareness of specific component(s) of physical activity guidelines for older adults**											
Do you know how many minutes of moderate intensity physical activity that the national guidelines recommend per week for older adults in your jurisdiction? Correct answer: 150	Yes	212	61.1	15	41.7	134	78.8	44	42.7	19	50.0
	Correctly answered **	170	49.0	9	25.0	120	70.6	30	29.1	11	28.9
	No	94	27.1	15	41.7	15	8.8	49	47.6	15	39.5
	Not stated	41	11.8	6	16.7	21	12.4	10	9.7	4	10.5

Do you know how many minutes of vigorous intensity physical activity that the national guidelines per week for older adults in your jurisdiction? Correct answer: 75	Yes	134	38.6	7	19.4	94	55.3	20	19.4	13	34.2
	Correctly answered **	95	27.4	2	5.6	76	44.7	12	11.7	5	13.2
	No	171	49.3	23	63.9	55	32.4	71	68.9	22	57.9
	Not stated	42	12.1	6	16.7	21	12.4	12	11.7	3	7.9

Do you know how many days per week that national guidelines in your jurisdiction recommend older adults perform strength, balance, and flexibility training Correct answer: at least 2 days per week	Yes	188	54.2	10	27.8	123	72.4	32	31.1	23	60.5
	Correctly answered**	179	51.6	9	25.0	117	68.8	28	27.2	22	57.9
	No	121	34.9	20	55.6	28	16.5	61	59.2	12	31.6
	Not stated	38	11.0	6	16.7	19	11.2	10	9.7	3	7.9

Correctly answered all 3 questions **		85	24.5	2	5.6	68	40.0	11	10.7	4	10.5
Awareness of resources											
I am aware of resources (i.e., online resources and toolkits) to facilitate my knowledge development and practice of discussions/ assessment /prescription of physical activity with patients as a part of routine care	Yes	165	47.6	4	11.1	109	64.1	34	33.0	18	47.4
	No	141	40.6	26	72.2	40	23.5	60	58.3	15	39.5
	Not stated	41	11.8	6	16.7	21	12.4	9	8.7	5	13.2


* Questions linked to ‘Knowledge’ domain of TDF ** Of those who replied ‘Yes’ and correctly recalled how many minutes/days when prompted.

**Table 3:** The COVID-19 pandemic: implications for older adults’ physical activity.

Survey Question	Answer	All Respondents		General Practice		Physiotherapy		Occupational Therapy		Nursing	
		*N*	%	*N*	%	*N*	%	*N*	%	*N*	%
Public health and social measures introduced to prevent the spread of COVID -19 have reduced older adults’ levels of physical activity	Agree	247	71.2	20	55.6	123	72.4	79	76.7	25	65.8
	Somewhat agree	52	15.0	5	13.9	27	15.9	11	10.7	9	23.7
	Neither agree nor disagree	6	1.7	2	5.6	1	0.6	2	1.9	1	2.6
	Somewhat disagree	6	1.7	3	3.8			2	1.9	1	2.6
	Disagree										
	Not stated	36	10.4	6	16.7	19	11.2	9	8.7	2	5.3
In light of the COVID-19 pandemic—do you think that health professionals can play an increased role in promoting physical activity to older adults?	Yes	283	81.6	24	66.7	145	85.3	87	84.5	27	71.1
	No	5	1.4	2	5.6	6	3.5	1	1.0	2	5.3
	Don’t know	21	6.1	3	8.3			6	5.8	6	15.8
	Not stated	38	11.0	7	19.4	19	11.2	9	8.7	3	7.9
Considering the COVID-19 pandemic—how likely are you to discuss physical activity with older adults as part of routine practice?	More likely	166	47.8	12	33.3	79	46.5	56	54.4	19	50.0
	Same as usual	128	36.9	15	41.7	64	37.6	35	34.0	14	36.8
	Less likely	16	4.6	3	8.3	7	4.1	3	2.9	3	7.9
	Not stated	37	10.7	6	16.7	20	11.8	9	8.7	2	5.3

## Data Availability

Data is contained within the article or supplementary material (Table S1).

## Supplementary Materials

The following are available online at https://www.mdpi.com/article/10.3390/ijerph18116064/s1, Table S1: Theoretical domains of healthcare professionals’ behaviour in assessment, discussion, and prescription of physical activity in routine practice.
