# Supplementary material for: Healthcare Professionals Promotion of Physical Activity with Older Adults: A Survey of Knowledge and Routine Practice
Source: Int J Environ Res Public Health. 2021 Jun 4;18(11):6064. doi: 10.3390/ijerph18116064 (PMC8200063; doi:10.3390/ijerph18116064)
Supplement: Supplementary file 1 [file ijerph-18-06064-s001.zip › ijerph-1205949-supplementary.pdf]

Table S1. Theoretical domains of healthcare professional's behaviour in assessment, discussion, and prescription of physical activity in routine practice

Theoretical Domains	Survey Question	Answer	All respondents		General practitioner		Physiotherapist		Occupational therapist		Nursing	
			N	%	N	%	N	%	N	%	N	%
**Assessment of patient's physical activity levels**												
**Social/Professional role and Identity**	As a part of routine practice, do you formally assess whether a patient is active or inactive (i.e., do you use any physical activity screening tools?)*	**Always**	47	13.5	2	5.6	34	20.0	34	20.0	4	10.5
		**Usually**	47	13.5	4	11.1	31	18.2	31	18.2	1	2.6
		**Sometimes**	45	13.0	3	8.3	23	13.5	23	13.5	6	15.8
		**Never**	169	48.7	21	58.3	61	35.9	63	61.2	24	63.2
		**Not Stated**	39	11.2	6	16.7	21	12.4	9	8.7	3	7.9
**Discussing physical activity with patients**												
**Social/Professional role and Identity**	As a part of routine practice, do you initiate conversations with patients about physical activity?**	**Always**	144	41.5	5	13.9	99	58.2	28	27.2	12	31.6
		**Usually**	113	32.6	17	47.2	43	25.3	38	36.9	15	39.5
		**Sometimes**	51	14.7	8	22.2	7	18.4	27	26.2	9	5.3
		**Never**	2	0.6					1	1.0	1	2.6
		**Not Stated**	37	10.7	6	16.7	19	11.2	9	8.7	3	7.9
**Skills**	I have received suitable training to initiate conversations with patients about physical activity	**Agree**	104	30.0	5	13.9	75	44.1	18	17.5	6	15.8
		**Somewhat agree**	79	22.8			45	26.5	23	22.3	11	28.9
		**Neither agree nor disagree**	42	12.1	9	25.0	13	7.6	11	10.7	9	23.7

		**Somewhat disagree**	34	9.8	3	8.3	11	6.5	19	18.4	1	2.6
		**Disagree**	50	14.4	13	36.1	6	3.5	22	21.4	9	23.7
		**Not stated**	38	11.0	6	16.7	20	11.8	10	9.7	2	5.3
**Social/Professional role and Identity**	Discussing physical activity with patients is part of my work as a healthcare professional	**Agree**	244	70.3	23	63.9	139	81.8	58	56.3	24	63.2
		**Somewhat agree**	51	14.7	5	13.9	10	5.9	30	29.1	6	15.8
		**Neither agree nor disagree**	10	2.9	1	2.8	2	1.2	3	2.9	4	10.5
		**Somewhat disagree**	4	1.2					3	2.9	1	2.6
		**Disagree**	1	0.3	1	2.8						
		**Not stated**	37	10.7	6	16.7	19	11.2	9	8.7	3	7.9
**Memory, Attention, Decision Processes**	Discussing physical activity with patients is easy to remember to do	**Agree**	164	47.3	6	16.7	105	61.8	36	35.0	17	44.7
		**Somewhat agree**	93	26.8	13	36.1	36	35.0	36	35.0	13	34.2
		**Neither agree nor disagree**	25	7.2	5	13.9	9	8.7	9	8.7	2	5.3
		**Somewhat disagree**	24	6.9	4	11.1	5	2.9	11	10.7	4	10.5
		**Disagree**	4	1.2	2	5.6			2	1.9		
		**Not stated**	37	10.7	6	16.7	20	11.8	9	8.7	2	5.3
**Belief about capabilities**	I am confident that I can discuss physical activity as part of routine practice with patients even when the patient is not motivated	**Agree**	123	35.4	8	22.2	78	45.9	27	26.2	10	26.3
		**Somewhat agree**	127	36.6	14	38.9	59	34.7	36	35.0	18	47.4
		**Neither agree nor disagree**	29	8.4	3	8.3	6	3.5	15	14.6	5	13.2
		**Somewhat disagree**	22	6.3	3	8.3	5	2.9	11	10.7	3	7.9

		**Disagree**	9	2.6	1	2.8	3	1.8	5	4.9		
		**Not stated**	37	10.7	7	19.4	19	11.2	9	8.7	2	5.3
**Belief about capabilities**	I am confident that I can discuss physical activity as part of routine practice with patients even when there is little time	**Agree**	102	29.4	2	5.6	69	40.6	19	18.4	12	31.6
		**Somewhat agree**	119	34.4	11	30.6	54	31.8	39	37.9	15	39.5
		**Neither agree nor disagree**	33	9.5	7	19.4	12	7.1	12	11.7	2	5.3
		**Somewhat disagree**	42	12.1	9	25.0	13	7.6	16	15.5	4	10.5
		**Disagree**	14	4.0	1	2.8	3	1.8	8	7.8	2	5.3
		**Not stated**	37	10.7	6	16.7	19	11.2	9	8.7	3	7.9
**Discussing physical activity with older adults**												
**Knowledge**	I am aware of how to initiate conversations about physical activity with older adults as a part of routine care	**Agree**	202	58.2	14	38.9	117	68.8	49	47.6	22	57.9
		**Somewhat agree**	88	25.4	12	33.3	30	17.6	32	31.1	14	36.8
		**Neither agree nor disagree**	11	3.2	3	8.3			8	7.8		
		**Somewhat disagree**	5	1.4	1	2.8	2	1.2	2	1.9		
		**Disagree**	3	0.9	12	33.3			3	2.9		
		**Not stated**	38	11.0	6	16.7	21	12.4	9	8.7	2	5.3
**Skills**	I have the skills to initiate conversations with older adults about physical activity	**Agree**	187	53.9	12	33.3	112	65.9	47	45.6	16	42.1
		**Somewhat agree**	91	26.2	13	36.1	30	17.6	34	33.0	14	36.8
		**Neither agree nor disagree**	20	5.8	2	5.6	7	4.1	6	5.8	5	13.2
		**Somewhat disagree**	10	2.9	2	5.6	2	1.2	5	4.9	1	2.6
		**Disagree**	3	0.9	1	2.8			2	1.9		

		**Not stated**	36	10.4	6	16.7	19	11.2	9	8.7	2	5.3
**Social/ Professional role and Identity**	As a healthcare professional it is my job to discuss physical activity with older adults	**Agree**	236	68.0	23	63.9	130	76.5	55	53.4	28	73.7
		**Somewhat agree**	55	15.9	3	8.3	16	9.4	29	28.2	7	18.4
		**Neither agree nor disagree**	10	2.9	1	2.8	1	0.6	7	6.8	1	2.6
		**Somewhat disagree**	4	1.2	1	2.8			3	2.9		
		**Disagree**	1	0.3	1	2.8						
		**Not stated**	41	11.8	7	19.4	23	13.5	9	8.7	2	5.3
**Belief about consequences**	For me, discussing physical activity with an older adult in routine practice is useful	**Agree**	193	55.6	15	41.7	115	67.6	44	42.7	19	50.0
		**Somewhat agree**	93	26.8	12	33.3	31	18.2	38	36.9	12	31.6
		**Neither agree nor disagree**	14	4.0	1	2.8	3	1.8	7	6.8	3	7.9
		**Somewhat disagree**	5	1.4	1	2.8	1	0.6	2	1.9	1	2.6
		**Disagree**	2	0.6	1	2.8			1	1.0		
		**Not stated**	40	11.5	6	16.7	20	11.8	11	10.7	3	7.9
**Intentions**	I intend to discuss physical activity in the next consultation/appointment with an older adult as part of routine care	**Agree**	191	55.0	10	27.8	43	41.7	117	68.8	21	55.3
		**Somewhat agree**	68	19.6	10	27.8	25	24.3	24	14.1	9	23.7
		**Neither agree nor disagree**	35	10.1	6	16.7	17	16.5	8	4.7	4	10.5
		**Somewhat disagree**	11	3.2	2	5.6	7	6.8	1	0.6	1	2.6
		**Disagree**	5	1.4	2	5.6	2	1.9			1	2.6

		**Not stated**	37	10.7	6	16.7	9	8.7	20	11.8	2	5.3
**Environmental context**	In the organisation that I work in, discussing physical activity with older patients is something that I typically do	**Agree**	170	49.0	6	16.7	114	67.1	34	33.0	16	42.1
		**Somewhat agree**	80	23.1	11	30.6	27	15.9	29	28.2	13	34.2
		**Neither agree nor disagree**	29	8.4	7	19.4	8	4.7	12	11.7	2	5.3
		**Somewhat disagree**	22	6.3	5	13.9	2	1.2	11	10.7	4	10.5
		**Disagree**	9	2.6					8	7.8	1	2.6
		**Not stated**	37	10.7	7	19.4	19	11.2	9	8.7	2	5.3
**Behavioural regulation**	Discussing physical activity in routine practice with older adults is something I do automatically	**Agree**	133	38.8	4	11.1	97	57.1	23	22.3	9	23.7
		**Somewhat agree**	100	28.8	12	33.3	34	20.0	33	32.0	21	55.3
		**Neither agree nor disagree**	32	9.2	8	22.2	11	6.5	11	10.7	2	5.3
		**Somewhat disagree**	31	8.9	3	8.3	8	4.7	17	16.5	3	7.9
		**Disagree**	14	4.0	3	8.3	1	0.6	9	8.7	1	2.6
		**Not stated**	37	10.7	6	16.7	19	11.2	10	9.7	2	5.3
**Behavioural regulation**	I have a clear plan of how I will initiate discussions about physical activity in routine practice with older adults	**Agree**	106	30.5	4	11.1	77	45.3	19	18.4	6	15.8
		**Somewhat agree**	92	26.5	7	19.4	44	25.9	27	26.2	14	36.8
		**Neither agree nor disagree**	52	15.0	7	19.4	18	10.6	19	18.4	8	21.1
		**Somewhat disagree**	46	13.3	8	22.2	11	6.5	21	20.4	6	15.8
		**Disagree**	14	4.0	4	11.1	1	0.6	7	6.8	2	5.3

		**Not stated**	37	10.7	6	16.7	19	11.2	10	9.7	2	5.3
**Use of physical activity guidelines, prescription, and signposting**												
**Environmental context**	Physical activity guidelines have a place in routine practice	**Agree**	224	64.6	18	50.0	117	68.8	61	59.2	28	73.7
		**Somewhat agree**	64	18.4	9	25.0	25	14.7	23	22.3	7	18.4
		**Neither agree nor disagree**	16	4.6	2	5.6	5	2.9	8	7.8	1	2.6
		**Somewhat disagree**	4	1.2			2	1.2	2	1.9		
		**Disagree**	1	0.3	1	2.8						
		**Not stated**	38	11.0	6	16.7	21	12.4	9	8.7	2	5.3
**Environmental context**	There is sufficient time allocated in day-to-day work (routine practice) to implement physical activity guidelines for adults/older adults	**Agree**	68	19.6	2	5.6	45	26.5	15	14.6	6	15.8
		**Somewhat agree**	91	26.2	3	8.3	55	32.4	23	22.3	10	26.3
		**Neither agree nor disagree**	50	14.4	6	16.7	19	11.2	18	17.5	7	18.4
		**Somewhat disagree**	68	19.6	8	22.2	25	14.7	26	25.2	9	23.7
		**Disagree**	33	9.5	11	30.6	7	4.1	11	10.7	4	10.5
		**Not stated**	37	10.7	6	16.7	19	11.2	10	9.7	2	5.3
**Social/Professional role and Identity**	As a part of routine practice, do you signpost patients to other physical activity services (i.e., exercise referral programmes/community-based physical activity initiatives?)***	**Always**	42	12.1	1	2.8	30	17.6	5	4.9	6	15.8
		**Usually**	84	24.2	2	5.6	53	31.2	23	22.3	6	15.8
		**Sometimes**	131	37.8	20	55.6	56	32.9	42	40.8	13	34.2
		**Never**	53	15.3	6	16.7	12	7.1	24	23.3	11	28.9
		**Not Stated**	37	10.7	7	19.4	19	11.2	9	8.7	2	5.3

**Social influence**	I am supported to use physical activity discussions/ assessment/ prescription in everyday practice	**Agree**	99	28.5	1	2.8	66	38.8	24	23.3	8	21.1
		**Somewhat agree**	83	23.9	2	5.6	53	31.2	18	17.5	10	26.3
		**Neither agree nor disagree**	63	18.2	10	27.8	19	11.2	22	21.4	12	31.6
		**Somewhat disagree**	44	12.7	7	19.4	9	5.3	22	21.4	6	15.8
		**Disagree**	21	6.1	10	27.8	3	1.8	8	7.8		
		**Not stated**	37	10.7	6	16.7	20	11.8	9	8.7	2	5.3
**Belief about consequences**	If I assess/ discuss/ prescribe physical activity with an older adult as part of routine practice it will benefit the public health agenda	**Agree**	238	68.6	24	66.7	127	74.7	61	59.2	26	68.4
		**Somewhat agree**	48	13.8	3	8.3	16	9.4	25	24.3	4	10.5
		**Neither agree nor disagree**	19	5.5	2	5.6	8	4.7	6	5.8	3	7.9
		**Somewhat disagree**	4	1.2	1	2.8			1	1.0	2	5.3
		**Disagree**	2	0.6					1	1.0	1	2.6
		**Not stated**	36	10.4	6	16.7	19	11.2	9	8.7	2	5.3

* χ^2 (3, N=308)=10.3, p <0.05

** χ^2 (3, N=310)=11.9, p <0.05

*** χ^2 (3, N=310)=18.6, p <0.001
